# American Institute of Homœopathy

**Published:** 1896-02

**Authors:** W. A. Dewey

**Affiliations:** Chairman, 170 W. Fifty-fourth Street, N. Y.


### AMERICAN INSTITUTE OF HOMŒOPATHY.

### TRANSPORTATION COMMITTEE, BULLETIN NO. 1.

The next meeting of the American Institute of Homœopathy will be held in Detroit, Mich., from Wednesday, June 17th, to Thursday, June 25th. The Materia Medica Conference, which promises to be a most interesting feature, will convene on Tuesday, June 16th, at 3 P. M. All interested in Materia Medica should be present.

There is every prospect that the usual rate of a fare and a third for the round trip will be allowed by the Traffic Associations. The Joint Traffic Association, which resulted from the consolidation of the Central Traffic Association and the Trunk Line Association, now controls all the territory between New York and Chicago. This association goes into effect February 1st, provided an injunction against its legality, brought by the Attorney-General, is not sustained. In the latter case, no one can tell what the outcome may be.

At the rate of a fare and a third, or regular convention rates, the fares from some of our principal points would be approximately: New York, $18.00; Boston, $22.65; Philadelphia,

Washington, and Baltimore, $19.00; Chicago, $10.33; St. Louis, $17.33, and Kansas City, $25.33.

The time from New York and Philadelphia is approximately 18 hours; from Boston, 20 hours; Chicago, 8 hours; St. Louis, 14 hours, and Kansas City, 23 hours.

Detroit is very centrally situated, is quite a railroad centre, and is very easily reached. From New York, one may take one of no less than fifteen routes, and in the West, especially at the time of the meeting, all roads should lead to Detroit.

The Michigan Central road and its branches cover most of the territory in the State of Michigan, as well as offering unexceptional facilities from Buffalo and Chicago. Michigan is credited with some five hundred homœopathic physicians, certainly seventy-five per cent. of these should be present at the meeting in Detroit.

The Chicago & Alton, and the Union Pacific roads, which treated the Institute so royally at the time of the Denver meeting, should not be overlooked by our Western friends, nor should the Chicago & North Western be forgotten by our Northwestern delegates.

The Lehigh Valley road offers a specially fine service for our Washington, Baltimore, and Philadelphia members; it runs in connection with the Grand Trunk Railway of Canada, which also covers points as far east as Boston, and as far west as Chicago.

The committee is constantly at work to obtain the best accommodations for the greatest number at the best possible rates, and monthly bulletins will keep the profession posted as to best routes, train services, and all railroad matters influencing the meeting. A large attendance should be present.

W. A. Dewey, M. D., *Chairman*,
170 W. Fifty-fourth Street, N. Y.

